# Direct Single-Molecule Observation of Sequential DNA Bending Transitions by the Sox2 HMG Box

**DOI:** 10.3390/ijms19123865

**Published:** 2018-12-04

**Authors:** Mahdi Muhammad Moosa, Phoebe S. Tsoi, Kyoung-Jae Choi, Allan Chris M. Ferreon, Josephine C. Ferreon

**Affiliations:** Department of Pharmacology and Chemical Biology, Baylor College of Medicine, Houston, TX 77030, USA; Mahdi.Moosa@bcm.edu (M.M.M.); Phoebe.Tsoi@bcm.edu (P.S.T.); Kyoungjae.Choi@bcm.edu (K.-J.C.)

**Keywords:** transcription factors, DNA-protein interactions, Sox2 sequential DNA loading, smFRET, DNA conformational landscape, sequential DNA bending, transcription factor dosage

## Abstract

Sox2 is a pioneer transcription factor that initiates cell fate reprogramming through locus-specific differential regulation. Mechanistically, it was assumed that Sox2 achieves its regulatory diversity via heterodimerization with partner transcription factors. Here, utilizing single-molecule fluorescence spectroscopy, we show that Sox2 alone can modulate DNA structural landscape in a dosage-dependent manner. We propose that such stoichiometric tuning of regulatory DNAs is crucial to the diverse biological functions of Sox2, and represents a generic mechanism of conferring functional plasticity and multiplicity to transcription factors.

## 1. Introduction

Sox2 regulates a remarkable variety of genes differentially; it activates some and represses others [1,2,3]. This functional diversity is assumed to be mediated by Sox2 heterodimerization with other transcription factors (TFs) such as Oct4, Oct1, Pax6, and Nanog [4,5]. Recent reports, however, suggest that these canonical partners often remain spatiotemporally separated from Sox2 during genome engagement [6,7,8,9,10]. This raises an important question regarding the TF’s mechanism of action as to how Sox2 alone can exert differential loci-specific regulatory effects.

Sox2 is a sequence-specific high-mobility group transcription factor (HMG-TF) [11]. These TFs have conserved DNA binding domains [12,13], also known as HMG box. These DNA binding domains are partly disordered and are assumed to undergo binding-induced functional disorder-to-order transitions [14]. HMG-TFs are known to cooperatively form heterodimers on DNA regulatory elements [13,15,16,17]; each heteromeric TF pair induces characteristic DNA bend and differentially regulates target gene transcription [18,19,20]. Interestingly, a number of recent studies suggested that Sox2 can also function as homodimers [21,22,23]. Whether and how such Sox2 assemblies alter DNA conformations remain largely unknown. Here, we utilize the strengths of single-molecule Förster/fluorescence resonance energy transfer (smFRET) measurements along with ensemble methods to understand the effects of Sox2 binding on regulatory DNA structural landscape in the context of the HMG box (Sox2^HMG^). Our results suggest that Sox2^HMG^ induces stoichiometry-dependent alternate DNA bends and we propose that the resulting alternate DNA conformations may drive different transcriptional outcomes. 

## 2. Results

### 2.1. Multiple Sox2^HMG^ Domains Cooperatively Interact with dsDNA^NANOG^

**Figure 1:**
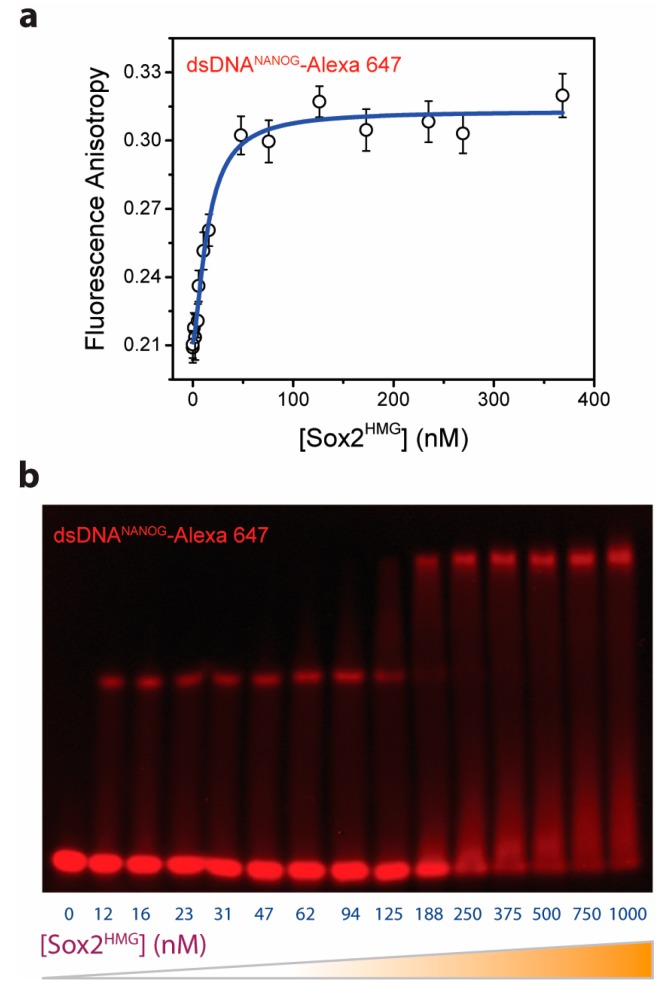
Sox2 cooperatively binds to the *NANOG* upstream promoter (DNA^NANOG^). (**a**) DNA binding of Sox2^HMG^ was probed by monitoring changes in fluorescence anisotropy of Alexa Fluor 647-labeled dsDNA with increasing [Sox2^HMG^]. The solid line represents nonlinear least squares (NLS) fit of the data to a Hill equation. NLS-derived parameters: *K*_D_ = 15.1 (±2.0) nM, Hill coefficient = 1.5 (±0.3). (**b**) Fluorescence electrophoretic mobility assay (fEMSA) of Sox2^HMG^-DNA^NANOG^ binding suggests a multistep Sox2^HMG^ complex formation with dsDNA^NANOG^ involving multiple protein molecules that are able to bind the DNA partner. (See also Figure S2.)

In our initial ensemble experiments, we observe that Sox2^HMG^ cooperatively binds to the *NANOG* composite promoter (DNA^NANOG^; Figure 1). We utilized fluorescence anisotropy to detect Sox2^HMG^ binding to dsDNA^NANOG^ (Supplementary Methods). Anisotropy reports on fluorophore rotational properties, dependent on both probe local and global environment perturbations; fluorescence anisotropy of labeled macromolecule usually increases upon ligand binding. To characterize Sox2^HMG^-DNA binding, we singly-labeled dsDNA^NANOG^ with Alexa Fluor 647 (Supplementary Methods) and monitored changes in DNA fluorescence anisotropy with increasing Sox2^HMG^ concentrations (Figure 1a). Nonlinear least squares (NLS) fitting of the anisotropy data to a Hill equation yields an apparent dissociation constant (*K*_D_) of 15.1 (±2.0) nM and Hill coefficient of 1.5 (±0.3). The estimated *K*_D_ is similar to that previously reported for specific DNA-Sox2 interactions [18]. A Hill coefficient greater than 1 indicates that multiple Sox2 HMG boxes bind to the DNA in a TF concentration-dependent fashion [24]. Anisotropy measurements also indicate that Sox2^HMG^ alone (i.e., the DNA-binding domain in the absence of dsDNA^NANOG^) fails to dimerize/oligomerize (Figure S1). To verify the binding of multiple Sox2 molecules to DNA^NANOG^, we carried out fluorescence electrophoretic mobility shift assay (fEMSA) of DNA with increasing [Sox2^HMG^]. The fEMSA micrograph shows concentration-dependent appearance of multiple electrophoretic species (Figure 1b). This suggests a multistep Sox2^HMG^ interaction with the *NANOG* proximal promoter. The non-equilibrium nature of mobility shift assays, however, precludes precise estimation of binding affinities of individual Sox2-DNA assemblies on the basis the fEMSA micrograph [25].

### 2.2. Sox2^HMG^ Induces Sequential dsDNA^NANOG^ Bending Transitions

Next, we focused on understanding the mechanism of the TF-DNA complex formation. Although the mobility shift assay clearly demonstrates a multistep higher-order Sox2^HMG^ complex formation with the dsDNA (Figure 1b), our ensemble experiments (i.e., fluorescence anisotropy and fEMSA) were not sensitive enough to determine the stoichiometries of respective TF-DNA complexes. To directly observe Sox2^HMG^-DNA^NANOG^ binding steps, we performed single-molecule fluorescence microscopy experiments that provide key advantages over conventional ensemble methods: (1) individual conformational sub-populations that are averaged out in ensemble measurements can be directly detected; and (2) experiments can be performed with extremely low concentrations of the labeled molecule (typically 50–100 pM). The ability to carry out experiments at low biomolecule concentration provides access and resolution for characterizing individual interaction steps in tightly interacting systems.

**Figure 2:**
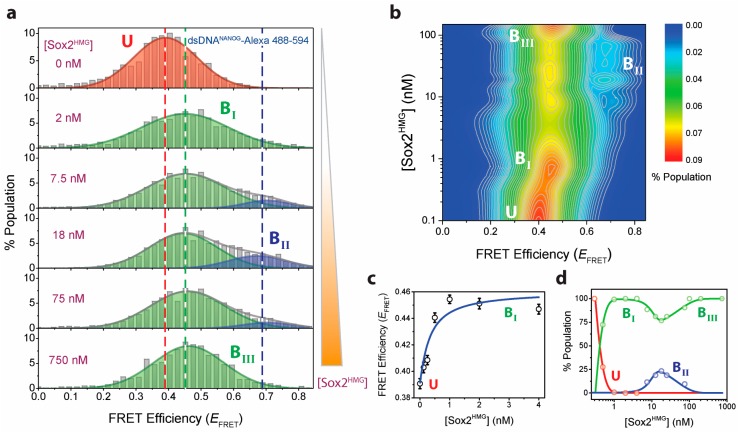
smFRET reveals Sox2^HMG^ concentration-dependent multistep bending of DNA^NANOG^. (**a**) *E*_FRET_ histograms of DNA^NANOG^ with increasing [Sox2^HMG^]. (**b**) [Sox2^HMG^]-*E*_FRET_ contour map color coded based on fractional occupancy of individual DNA conformations. Corresponding DNA conformations are marked on the contour map. (**c**) Sox2 binding isotherm of the U ⇋ B_I_ transition as probed by detecting changes in *E*_FRET_, linked to dsDNA bending transition. The NLS-derived apparent *K*_D_ for this binding step is 0.30 (±0.04) nM (binding equation with fixed Hill coefficient of 1). (**d**) dsDNA^NANOG^ conformational distributions as modulated by Sox2^HMG^ concentration, determined from NLS fitting of individual smFRET histograms to Gaussian functions.

We utilized the distance-dependence of FRET to characterize Sox2^HMG^-DNA^NANOG^ interaction at single-molecule resolution. smFRET is sensitive to distance changes in the 20–70 Å range [26], and provides the necessary spatial resolution to probe changes in dsDNA^NANOG^ conformations as induced by TF binding (estimated end-to-end distance of DNA^NANOG^ is 57.4 Å, assuming inter-base axial rise of 3.4 Å [27]). For the smFRET experiments, we labeled DNA^NANOG^ with Alexa Fluor 488 and 594 donor-accepter dye-pair (Supplementary Methods). Bursts of fluorescence from donor and acceptor dyes were recorded as dual-labeled *NANOG* promoter DNA passed through the sub-fL observation volume of our custom-built ISS Alba confocal laser microscopy system (described previously [28]). These fluorescence intensities were converted to FRET efficiency (*E*_FRET_) histograms, providing a scheme for direct visualization of DNA conformational distributions. Without Sox2^HMG^, the dual-labeled DNA showed a single-peak in its *E*_FRET_ histogram with histogram width typical of smFRET studies of freely diffusing dsDNA molecules [29,30] (Figure 2a; top panel). An NLS fit of the histogram to a Gaussian function yielded *E*_FRET_ value of 0.39 (±0.04). On the basis of this *E*_FRET_ value, we estimate the apparent distance between the two dyes to be approximately 64.6 Å (assuming a Förster distance of 60 Å between Alexa 488/594 dyes [31]). This is consistent with the estimated end-to-end distance of dsDNA^NANOG^, where the slight increase in the apparent distance (compared to the estimated distance) can be attributed to the linkers present in Alexa dyes.

Often, histograms of data collected in diffusion-based smFRET experiments show an additional peak at zero *E*_FRET_ that arise from molecules with active donor(s) and either inactive or absent acceptor [29,30,32,33,34,35]. These zero *E*_FRET_ peaks tend to significantly overlap with low *E*_FRET_ peak populations and hamper direct estimation of the position of the non-zero peak(s) [36,37,38,39]. Interestingly, our smFRET histograms lack zero *E*_FRET_ peaks (Figure 2a). We attribute this to the absence of dual donor-labeled dsDNA molecules as ensured by sequential labeling of individual DNA strands (Supplementary Methods). Therefore, sequential labeling and purification of individual fluorophore-conjugated oligos prior to duplex formation can be utilized to minimize zero peaks.

DNA bending (also known as DNA looping) is critical for many eukaryotic TF function [40,41,42,43,44]. Accordingly, Sox2 was shown to induce binding-mediated *FGF* (fibroblast growth factor) enhancer bending [18]. We postulate that similar spatially precise bending is induced in Sox2-DNA^NANOG^ complexes during gene regulation. To characterize Sox2^HMG^ binding-induced *NANOG* promoter DNA bending, we carried out isothermal smFRET Sox2^HMG^ titration against approximately 100 pM dual-labeled DNA (Figure 2). Our smFRET experiments provide a direct way to distinguish between subtle conformational changes of DNA^NANOG^ induced upon Sox2 binding. In our smFRET experiments, we observed a multistep bending transition in the DNA structural landscape (Figure 2a). Initially, DNA^NANOG^ undergoes a cooperative bending to a 0.45 (±0.01) *E*_FRET_ state that corresponds to 32.1° (±1.4°) apparent bend angle at low Sox2^HMG^ concentrations (≤4 nM) (see Supplementary Methods for the details of FRET-to-apparent-angle conversion). NLS fit of the data yields an estimated *K*_D_ of 305 (±39) pM (Figure 2c). Such a tight interaction is unlikely to be driven by higher order Sox2^HMG^ assemblies and we therefore postulate that this dsDNA conformation (henceforth referred as B_I_) is induced by binding to single Sox2^HMG^ molecules.

Our ensemble results suggested that multiple Sox2^HMG^ can form higher order TF-DNA assemblies (Figure 1). To characterize the complex formation, we probed for changes in DNA^NANOG^ conformations upon further addition of Sox2 on preformed monomeric Sox2^HMG^-DNA^NANOG^ complexes. With increasing [Sox2^HMG^], we observe a progressive reduction of the B_I_ population and the emergence of a new population exhibiting higher *E*_FRET_ (~0.68). This higher *E*_FRET_ population corresponds to a DNA^NANOG^ apparent bend angle of 70° (±2.4°; henceforth referred to as B_II_ DNA conformation). We infer that this DNA conformation is induced by sequential binding of two individual Sox2 TFs on the dsDNA, where binding of each monomer induces an approximate 32° bend at respective binding sites. Our observed apparent bend angle in the ternary complex (two Sox2 monomers and DNA) is similar to the DNA bend angle previously resolved for heterodimeric HMG box TF-DNA complexes [11,17].

Interestingly, an additional transition is visible in our isothermal smFRET titration when additional Sox2^HMG^ is added (i.e., >75 nM [Sox2^HMG^]). We observe progressive depopulation of the B_II_ bent DNA conformation and coupled emergence of a population at *E*_FRET_ ~0.44 as [Sox2^HMG^] increases further (henceforth referred as B_III_; Figure 2a). We estimate the apparent bend angle for the B_III_ population to be 30.4° (±4.5°) from the *E*_FRET_ data (Supplementary Methods). A longer fEMSA run also indicates higher-order oligomer formation that is consistent with the formation of B_III_ population (Figure S2). Mechanistically, Sox family TFs induce DNA bends via FM dipeptide intercalation between two Thymine (T) bases at the minor groove interface [45,46]. Within the *NANOG* composite promoter, three TT pairs are present: two within the two HMG-TF binding sites (Oct/Sox motifs) identified by Rodda et al. [47] and one in between. We hypothesize that the initial two DNA bends are induced by sequential Sox2 binding to the two high-affinity HMG-TF binding motifs, where each binding induces an apparent 32° bend at the sites of interactions (a net 70° DNA apparent bend angle in the ternary complex). As Sox2^HMG^ concentration further increases (>75 nM), an additional TF molecule interacts with the DNA at the remaining TT site and induces similar bend albeit at the opposite DNA face. This results in effective reversal of the second bend as evidenced by the increased inter-dye distance (i.e., reduced *E*_FRET_) at higher [Sox2^HMG^]. The final bend remains relatively unchanged upon further increase in Sox2 (up to 1 µM; Figure 2d). Overall, our smFRET data directly demonstrates multistep sequential DNA bending transitions dependent on Sox2 concentration.

## 3. Discussion

**Figure 3:**
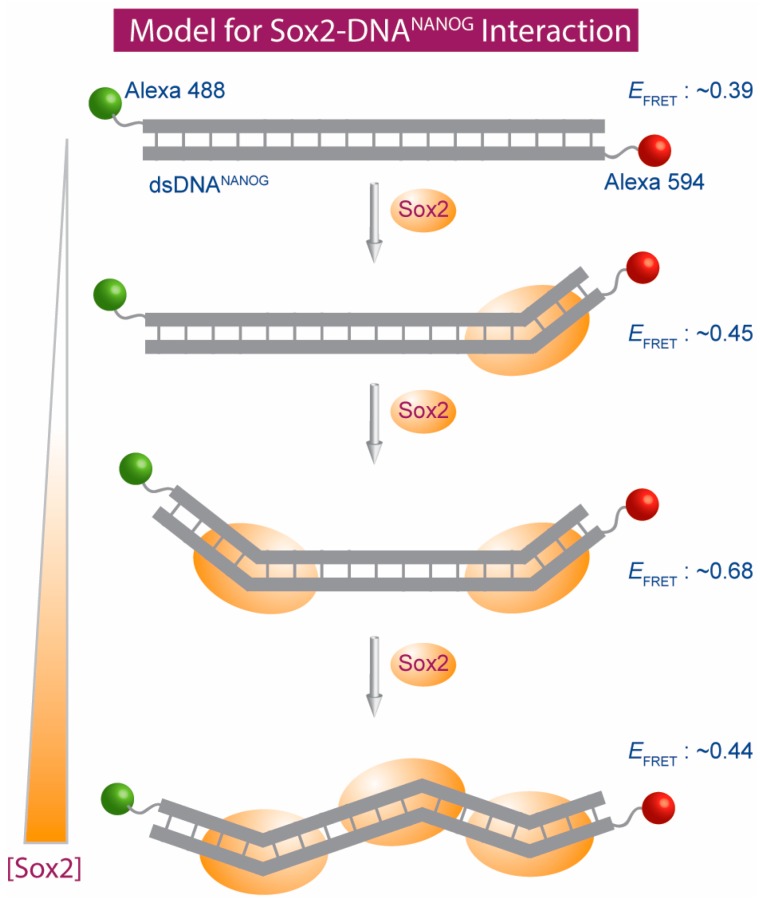
Schematic representation of the Sox2 stoichiometry-dependent dsDNA bending transitions.

Sox2 is a tightly regulated transcription factor; both significant increases and decreases in Sox2 dosage can be detrimental to its biological function [48,49]. Alterations in Sox2 dosage result in multiple developmental and acquired disorders [50,51,52,53,54]. We show that the Sox2 HMG box can induce concentration-dependent alternate DNA bends (Figure 3). Alternate promoter bends are likely to regulate genes differentially and initiate downstream cascades crucial for Sox2’s diverse functions. Our results provide a mechanism for Sox2’s strict dosage dependence in its function-dysfunction dichotomy [50,55,56,57,58].

In summary, our smFRET experiments clearly demonstrate the role of Sox2 dosage in modulating the conformational landscape of HMG box-binding DNA motifs. Previous studies on Sox family members suggested that heterodimeric homeodomain TFs can induce sequential bending as they interact with their DNA partners [59,60,61,62]. Here, we utilize the strengths of smFRET to demonstrate that a representative sequence-specific HMG-TF alone induces concentration-dependent multistep DNA bending transitions. We envision additional layers of tunability for heteromeric HMG-TFs in respective regulatory complexes where affinities of individual transcription factors for DNAs as well as inter-TF interactions can vary dramatically.

## 4. Materials and Methods

Experimental details are provided in the Supplementary Materials. Briefly, ensemble fluorescence anisotropy and fluorescence electrophoretic mobility assay (fEMSA) experiments were performed in Buffer E (20 mM Tris, 50 mM NaCl, 0.10 mg/mL BSA, 5% glycerol, 0.1 mM DTT/0.05 mM TCEP, pH 8) with Alexa Fluor-647 labeled dsDNA^NANOG^ (Forward: ACTTTTGCATTACAATG; 17 bp). smFRET experiments were performed in the same buffer using a custom-built confocal fluorescence microscopy set up as described previously [28].

## Supplementary Materials

Supplementary materials can be found at http://www.mdpi.com/1422-0067/19/12/3865/s1.

## Abbreviations

fEMSA	fluorescence electrophoretic mobility shift assay
FGF	fibroblast growth factor
HMG	high mobility group
NLS	nonlinear least squares
smFRET	single-molecule Förster resonance energy transfer
TF	transcription factor
