# Supplementary material for: Direct Single-Molecule Observation of Sequential DNA Bending Transitions by the Sox2 HMG Box
Source: Int J Mol Sci. 2018 Dec 4;19(12):3865. doi: 10.3390/ijms19123865 (PMC6321608; doi:10.3390/ijms19123865)
Supplement: Supplementary file 1 [file ijms-19-03865-s001.pdf]

Supplementary Figures

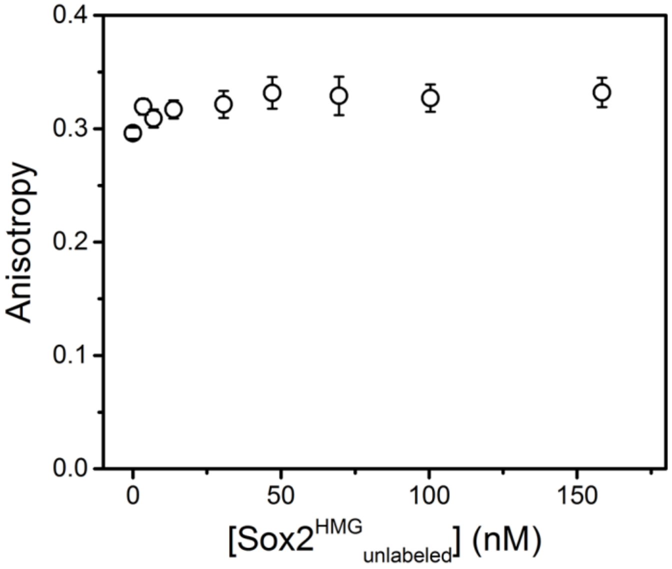

Figure S1. Sox2^{HMG} remains monomeric in the absence of dsDNA. Isothermal ensemble fluorescence anisotropy measurements on Alexa 594-labeled Sox2^{HMG} performed in increasing amounts of unlabeled protein. Fluorescence anisotropy remains approximately constant (within experimental error limits) as function of unlabeled Sox2^{HMG} concentration. Polarization experiments were performed in Buffer E using 42 nM Alexa Fluor 594-labeled Sox2^{HMG} (*see Supplementary Methods*).

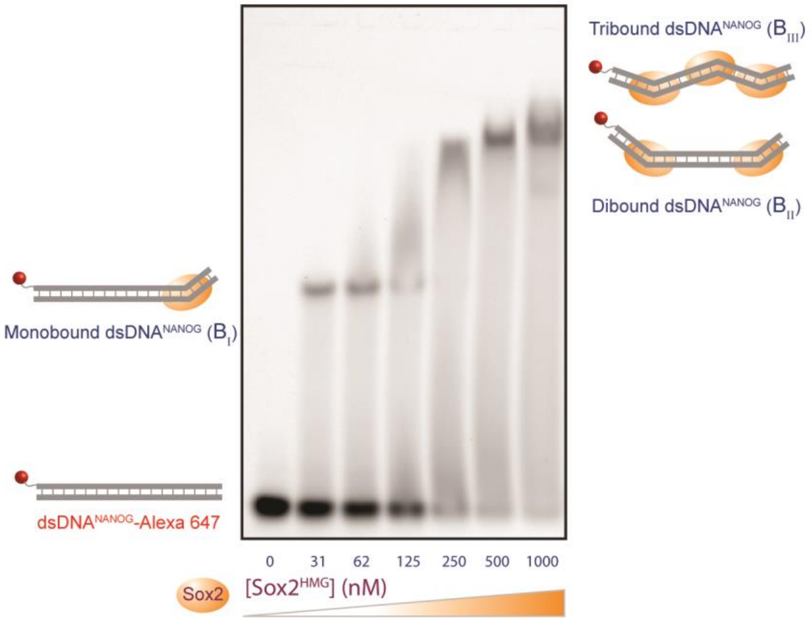

Figure S2. DNA^{NANOG}-Sox2^{HMG} interaction monitored by fluorescence electrophoretic mobility shift assay (fEMSA). A longer running time as compared to the fEMSA data presented in Figure 1b provides evidence for higher order oligomer formation. An increase in streaking at 1000 nM Sox2^{HMG} is consistent with formation of DNA^{NANOG}-(Sox2^{HMG})$_3$ quaternary complex, hypothesized on the basis of the smFRET binding experiments (Figure 2). fEMSA conditions: 5% TBE gel in Buffer E for 1 hr at 120 V, 4°C .

Supplementary Methods

Cloning, Protein Expression and Purification

The backbone vector was prepared by introducing DNA sequences of the streptococcal protein G B1 domain (GB1), TEV (tobacco etch virus) protease cleavage site, a tetracysteine tag (FLNCCPGCCMEP), and a thrombin cleavage site into EcoRI/BamHI sites of pET302/NT-His (Invitrogen). The gene coding for Sox2 41-120 residues ($Sox2^{HMG}$) was custom synthesized by GeneArt Gene Synthesis (ThermoFisher Scientific, Waltham, MA, USA) with codon optimization for *Escherichia coli*, and sub-cloned into the AvrII/BamHI sites of the backbone vector. The constructed plasmid was transformed into BL21 Star (DE3). *E. coli* cells were grown at 37°C in Terrific Broth medium with the antibiotic carbenecillin. Cells were induced with 1 mM isopropyl β-D-1-thiogalactopyranoside (IPTG) after reaching OD_{600} of 0.8-1.0 and grown overnight at 18°C.

His_6-GB1 tagged $Sox2^{HMG}$ was purified using FPLC under denaturing conditions (50 mM Tris, 150 mM NaCl, 6 M Urea, pH 8) with a TALON His-tag column (GE, Marlborough, MA, USA). The protein was eluted with 200 mM Imidazole. The tagged protein was further purified by reverse-phase HPLC (Agilent, Santa Clara, CA, USA) and lyophilized. The lyophilized protein was dissolved in 50mM Tris, pH 8 buffer and incubated overnight with thrombin protease (1U/mg; GE) at 30°C. Cleaved $Sox2^{HMG}$ was then purified by reverse-phase HPLC, lyophilized and frozen at -80°C until further use.

DNA/Protein Labeling

NANOG promoter DNA oligos (Forward: ACTTTTGCATTACAATG; 17 bp) with 5′ amino modifier C6 (IDT) were purified by ethanol precipitation and labeled using 10-fold molar ratio of dyes (Alexa 488, Alexa 594 or Alexa 647 NHS ester; Invitrogen) to DNA. The labeling reactions were performed at 30°C (0.5-1 hr incubation). Oligos were ethanol-precipitated from the labeling reaction mix and collected DNA pellets were dissolved in 0.1 M TEAA (triethylammonium acetate), pH 7. Reverse-phase HPLC was performed to separate unlabeled oligos. For smFRET experiments, the Alexa 488-labeled forward oligo was annealed to Alexa 594-labeled reverse oligo, applying a slow temperature gradient (0.29°C/min) from 95°C to room temperature using a thermal cycler. $Sox2^{HMG}$ was labeled with Alexa fluor 594 NHS ester (Invitrogen, Carlsbad, CA, USA).

Ensemble Fluorescence Spectroscopy

Fluorescence anisotropy experiments were carried out with 5 nM Alexa Fluor 647-labeled DNA^{NANOG} using an ISS PC1 photon-counting spectrofluorometer equipped with a Peltier automated temperature control unit (ISS Inc., Champaign, IL, USA). Anisotropy measurements were collected using excitation and emission wavelengths of 611 nm and 667 nm, respectively. Error bars are standard deviations from 30 measurements. Anisotropy experiments were carried out at 25°C in Buffer E (20 mM Tris, 50 mM NaCl, 0.10 mg/mL BSA, 5% glycerol, 0.1 mM DTT/0.05 mM TCEP, pH 8).

Fluorescence Electrophoretic Mobility Shift Assay (fEMSA) of $Sox2^{HMG}$: DNA^{NANOG} Binding

The fEMSA binding reactions consisted of 5 nM Alexa 647- labeled DNA and 11.7 nM -1 μM $Sox2^{HMG}$ in Buffer E. The samples were loaded on a 5% pre-cast Mini-PROTEAN Tris-Borate-EDTA (TBE, Bio-Rad, Hercules, CA, USA) gel and run for 50 min at 120 mV, 4°C, in TBE buffer. The gel was imaged using ChemicDoc™ with an Alexa 647 filter.

Single-Molecule FRET

Single-molecule FRET experiments were performed at room temperature using a custom-built ISS Alba confocal microscope (ISS Inc.) described previously (1). All isothermal single-molecule experiments were performed in Buffer E at room temperature. Donor and acceptor fluorescence were recorded at ~100 pM concentrations of Alexa Fluor 488/594-labeled $dsDNA^{NANOG}$. The binning time for individual experiments was 500 μs. The leakage of donor emission into the acceptor channel (1%) and acceptor emission due to direct excitation (3%) were taken into account. A lower threshold of 50 and upper threshold of 700 counts were used, where threshold is the sum of signals from the donor and acceptor channels, within the binning time. FRET efficiencies (E_{FRET}) were calculated from the corrected donor (I_D) and acceptor (I_A) fluorescence intensities as

$$E_{\mathrm{FRET}} = \frac{I_{\mathrm{A}}}{I_{\mathrm{A}} + \gamma I_{\mathrm{D}}} \tag{1}$$

A value of unity was used for γ. FRET efficiency histograms were generated using VistaVision 4.1 (ISS, Inc.). Histograms were fitted with Gaussian functions in OriginPro 2016 (OriginLab, Northampton, MA, USA).

Calculation of DNA Bend Angle from FRET Efficiencies

Distance between the two dyes were estimated from E_{FRET} from the following relation:

$$R = \frac{R_O}{\left(\frac{1}{E_{\mathrm{FRET}}} - 1\right)^6} \tag{2}$$

Where R_0 is the Förster distance between the donor-acceptor dye-pair. Angles were calculated from the estimated distances. Because the end-to-end distance of our 17 bp DNA is significantly less than the persistence length of dsDNA (2), we assume that in the absence of interactions, $\mathrm{DNA}^{\mathrm{NANOG}}$ is linear (*i.e.,* has a bend angle of 0° and length l = 64.6 Å as estimated from experimentally determined E_{FRET} for $\mathrm{DNA}^{\mathrm{NANOG}}$ utilizing Equation (2)). For simplicity, we assumed that the TF binding-induced DNA bend is induced at the center of the DNA (3). Interaction-induced bend angles were estimated utilizing the following mathematical descriptions,

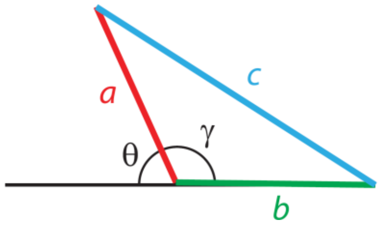

$$c = \sqrt{a^2 + b^2 - 2ab\cos\gamma} \tag{3}$$

$$\theta = 180° - \gamma \tag{4}$$

Supplementary References

1. Tsoi PS, Choi KJ, Leonard PG, Sizovs A, Moosa MM, MacKenzie KR, Ferreon JC, Ferreon ACM. The N-Terminal Domain of ALS-Linked TDP-43 Assembles without Misfolding. Angew Chem Int Ed Engl. 2017;56(41):12590-3. doi: 10.1002/anie.201706769. PubMed PMID: 28833982.
2. Brunet A, Tardin C, Salomé L, Rousseau P, Destainville N, Manghi M. Dependence of DNA Persistence Length on Ionic Strength of Solutions with Monovalent and Divalent Salts: A Joint Theory–Experiment Study. Macromolecules. 2015;48(11):3641-52. doi: 10.1021/acs.macromol.5b00735.
3. Coats JE, Lin Y, Rueter E, Maher LJ, 3rd, Rasnik I. Single-molecule FRET analysis of DNA binding and bending by yeast HMGB protein Nhp6A. Nucleic Acids Res. 2013;41(2):1372-81. doi: 10.1093/nar/gks1208. PubMed PMID: 23221634; PMCID: PMC3554232.
